# Neuronal Expression of Muscle LIM Protein in Postnatal Retinae of Rodents

**DOI:** 10.1371/journal.pone.0100756

**Published:** 2014-06-19

**Authors:** Evgeny Levin, Marco Leibinger, Anastasia Andreadaki, Dietmar Fischer

**Affiliations:** Division of Experimental Neurology, Department of Neurology, Heinrich-Heine-University Düsseldorf, Düsseldorf, Germany; Eye Hospital, Charité, Germany

## Abstract

Muscle LIM protein (MLP) is a member of the cysteine rich protein family and has so far been regarded as a muscle-specific protein that is mainly involved in myogenesis and the organization of cytoskeletal structure in myocytes, respectively. The current study demonstrates for the first time that MLP expression is not restricted to muscle tissue, but is also found in the rat naive central nervous system. Using quantitative PCR, Western blot and immunohistochemical analyses we detected MLP in the postnatal rat retina, specifically in the somas and dendritic arbors of cholinergic amacrine cells (AC) of the inner nuclear layer and the ganglion cell layer (displaced AC). Induction of MLP expression started at embryonic day 20 and peaked between postnatal days 7 and 14. It subsequently decreased again to non-detectable protein levels after postnatal day 28. MLP was identified in the cytoplasm and dendrites but not in the nucleus of AC. Thus, retinal MLP expression correlates with the morphologic and functional development of cholinergic AC, suggesting a potential role of this protein in postnatal maturation and making MLP a suitable marker for these neurons.

## Introduction

The family of cysteine rich proteins (CRP) comprises three closely related members, CRP1, CRP2, and CRP3. CRP3 is also known as muscle LIM protein (MLP) and has been postulated as a muscle-specific protein (mainly expressed in the heart). It is involved in the myogenesis and cytoskeletal organization of myocytes [1]–[5]. Accordingly, MLP deficiency leads to myocardial hypertrophy followed by cardiomyopathy and heart failure [6], [7]. In myocytes, MLP is localized in the cytoplasm, where it is involved in cytoskeleton modulation [4]. In addition, MLP has been reported to translocate to the nucleus to modulate gene expression during myogenesis and as part of a biomechanical stress response [8], [9]. A functional role of MLP in other tissues such as the central nervous system, such as the retina has not yet been described.

Amacrine cells (AC) are a heterogeneous group of retinal interneurons and synaptically interact with bipolar cells, retinal ganglion cells (RGCs) and other AC. Mammalian AC have been classified into more than 26 morphologic subtypes [10], [11] that are distinguishable by specific molecular markers, morphology, size and their neurotransmitters [12]. Cholinergic AC produce the neurotransmitter acetylcholine [13] allowing their specific identification by antibodies against choline acetyltransferase (ChAT) [14]. In rodents, ChAT-immunoreactive AC are detected from embryonic day 17 and produce acetylcholine lifelong [15]. AC are either conventionally located in the inner nuclear layer (INL) at the border with the inner plexiform layer (IPL) or displaced to the ganglion cell layer (GCL) [13], [16], [17]. The cells of the displaced subpopulation are stimulated by light and therefore termed ON-cells. Their dendrites are restricted to the ON sublamina of the IPL, whereas the AC located in the INL are excited in the absence of light (OFF cells). These AC accumulate their dendrites in the OFF-sublamina. As a consequence of this strict stratification pattern, two distinct ChAT-immunoreactive dendritic layers of cholinergic AC are visible in the IPL [18]. Moreover, cholinergic AC induce spontaneous waves of action potentials in the developing retina, which reportedly facilitates the formation of visual circuits between retinal neurons. This process is apparently driven by the release of acetylcholine [19], [20].

In mammals, the morphology of cholinergic AC proceeds development until the animals open their eyes between postnatal days 13–15 [15], [21]. Concurrently, AC form synapses with direction-selective RGCs during the first two postnatal weeks to establish functional circuits [22]. Islet1, NeuroD or Math3 are reportedly among the few so far identified factors that promote genesis and differentiation of AC [23], [24], while cell adhesion and guidance molecules such as semaphorins participate in the specific laminar stratification of AC [25], [26].

The current study reports that MLP is expressed in the cytoplasm of cholinergic AC during the late embryonic and the postnatal maturation stage, thereby demonstrating that MLP is also markedly expressed in other tissue than muscle and that MLP is a specific marker for postnatal cholinergic AC with a potential role in AC maturation.

## Materials and Methods

All experimental animal procedures were approved by the local animal care committee in Recklinghausen and conducted in compliance with federal and state guidelines for animal experiments in Germany (Permit Number: 84-02.04.2012.A300). Rats were maintained on a 12 hour light/dark cycle with ad libitum access to food and water. Rats were killed either by inhalation of CO_2_ or intraperitoneal application of ketamine (60–80 mg/kg; Pfizer) and xylazine (10–15 mg/kg; Bayer) and perfused through the heart with cold PBS (Gibco) followed by paraformaldehyde (Sigma) (4% PFA in PBS).

### RNA Isolation and Quantitative Real-time PCR

Total RNA was isolated from retinae of rats using the RNeasy kit (Qiagen) according to the manufacturer’s protocol. Retina-derived RNA (10 ng) of 4 different animals per age group was combined and reversely transcribed using the superscript II kit (Invitrogen). The cDNA quantification of MLP and glyceraldehyde 3-phosphate dehydrogenase (GAPDH) expression was performed with the SYBR Gree PCR Master Mix (Applied Biosystems) and QuantiTect primers (Rn_Csrp3_1_SG, Rn_Gapdh_1_SG QuantiTect Primer Assay (200); Qiagen) using the Real-Time PCR System (Applied Biosystems 7500). Retina-derived cDNA was amplified during 45 cycles according to the manufacturer’s protocol. All reactions were performed in duplicate and as two independent runs. Quantitative analysis was performed using Applied Biosystems 7500 software, calculating the expression of MLP relative to the endogenous housekeeping gene GAPDH. Relative quantification was calculated using comparative threshold cycle method (ΔΔCt). The specificity of the PCR products from each run was determined and verified with the dissociation curve analysis feature of the Applied Biosystems 7500 software. The significance in pairwise comparisons between age groups was evaluated using Mann-Whitney Rank Sum Test.

### Western Blot

For retinal lysate preparation, rat retinae were dissected and collected in lysis buffer (20 mM Tris/HCl (Sigma), pH 7.5, 10 mM KCl (AppliChem), 250 mM sucrose (Sigma), 10 mM NaF (Sigma), 1 mM DTT (Sigma), 0.1 mM Na_3_VO_4_ (Sigma), 1% Triton X-100 (Sigma), 0.1% SDS (Sigma)) with protease inhibitors (Calbiochem). Retinae were homogenized by sonification and centrifuged at 5000 rpm for 10 min. The supernatants obtained from 4 different animals per group were combined and used for western blot analysis. Separation of proteins was performed by 10% SDS polyacrylamide gel electrophoresis, according to standard protocols (Bio-Rad). Afterwards, proteins were transferred to polyvinylidene fluoride (PVDF) membranes (Bio-Rad). The blots were blocked in 5% dried milk with 1% bovine serum albumin (BSA) in Tris-buffered saline-Tween-20 and processed for immunostaining with either a polyclonal antibody against MLP (C9001-23; United States Biological; 1∶500) or a monoclonal antibody against β-actin (AC-15; Sigma; 1∶5000). Bound anti-MLP-antibody was visualized with anti-goat immunoglobulin G (IgG) secondary antibody conjugated to horseradish peroxidase (Sigma, 1∶30 000) and the antigen–antibody complexes were detected by enhanced chemiluminescence (Bio-Rad). Bound anti-actin-antibody was visualized with anti-mouse infrared dye secondary antibody (IRDey 800 CW; LI-COR; 1∶20 000) using Odyssey Infrared Imaging System (LI-COR).

### Immunohistochemistry and Quantification of MLP-positive AC

Rats were anesthetized and perfused through the heart with cold PBS followed by paraformaldehyde (4% PFA in PBS). Eyes were enucleated, post-fixed for several hours in 4% PFA, transferred to 30% sucrose overnight (4°C), and embedded in Tissue-Tek (Sakura). Frozen sections were cut longitudinally on a cryostat, thaw-mounted onto coated glass slides (Superfrost plus, Fisher, Pittsburgh, PA, USA) and stored at −20°C until further use. A polyclonal antibody against ChAT (AB144P; Merck Millipore; 1∶100) and a monoclonal antibody against MLP (a kind gift from Dr. Geier, Max Delbrück Center for Molecular Medicine, Berlin, Germany; 1∶400) were used. Anti-goat IgG and anti-mouse IgG antibodies conjugated to Alexa Fluor 488 and Alexa Fluor 594, respectively, were used as secondary antibodies (Invitrogen; 1∶1000). Stained sections were embedded with Mowiol (Calbiochem), covered using glass coverslips (VWR) and analyzed under a fluorescent microscope (Axiovision, Zeiss). For quantification of MLP-positive cholinergic AC, an intensity threshold was set to identify the positively stained cells. The percentage of MLP-expressing cholinergic AC was calculated as the ratio of MLP-positive to ChAT-positive cell numbers. Three animals per postnatal age and four sections per animal were quantified. Confocal images of fluorescent sections were obtained using confocal laser scanning microscope LSM 510 (Zeiss).

For retinal whole mount staining, rats were killed by decapitation and the retinae were rapidly dissected from the eyecups. Retinae were attached to nitrocellulose filter, fixed in 4% PFA for 30 min and treated with 2% TritonX (Sigma) for 1 h. The whole mounts were stained with antibodies against ChAT (1∶100) and MLP (1∶400), embedded with Mowiol and covered using glass coverslips. Confocal images were obtained using confocal laser scanning microscope LSM 510 (Zeiss).

### Dissociated AC Culture

Tissue culture plates (four-well-plates; Nunc) were coated with poly-D-lysine (0.1 mg/ml, molecular weight 300 000 Da; Sigma), rinsed with distilled water and air-dried. To prepare AC cultures, postnatal rats were killed by decapitation, retinae were rapidly dissected from the eyecups and incubated at 37°C for 30 min in a digestion solution containing papain (10 U/ml; Worthington) and L-cysteine (0.3 mg/ml; Sigma) in Neurobasal (NB) medium (Gibco). They were then rinsed with NB medium and triturated in 1 ml NB medium. Dissociated cells were passed through a cell strainer (40 mm) and 300 µl cell suspension in NB medium containing B27 supplement (1:50; Gibco), penicillin/streptomycin (0.2 mg/ml; Biochrom) and L-Glutamine (0.5 mM; Sigma) were added to each well. Cells remained in culture for 2 hours or 2 days and were fixed in 4% paraformaldehyde (PFA) solution in PBS for 25 min and then in 100% methanol (Sigma) for 10 min. AC were stained with antibodies against ChAT (1∶100) and MLP (1∶400).

## Results

### MLP Expression in Embryonic and Postnatal Rat Retina

**Figure 1:**
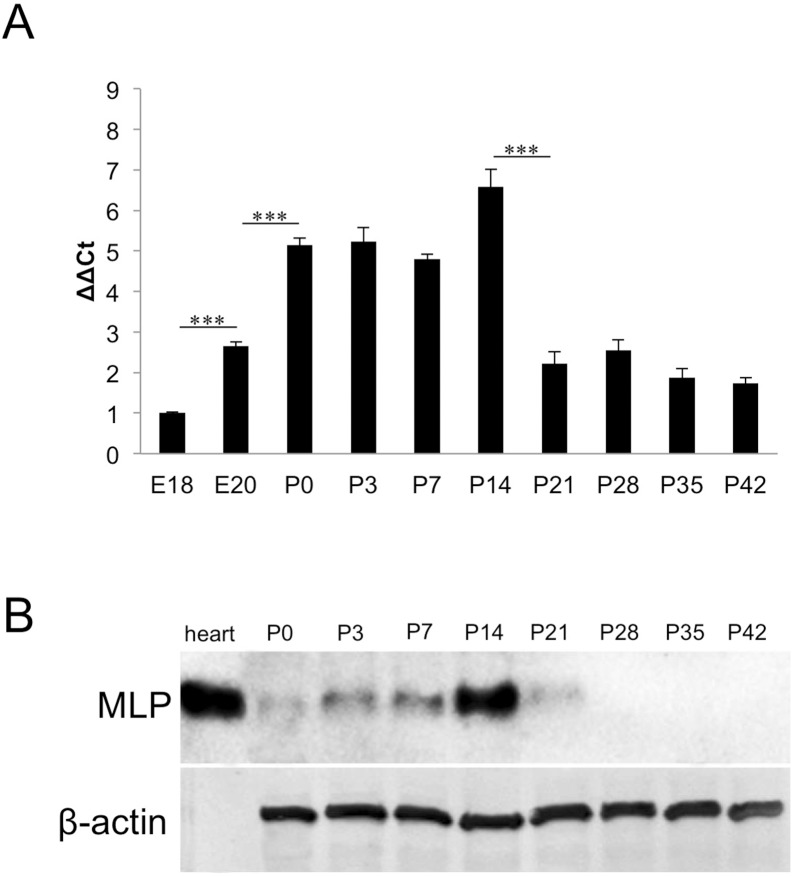
Transient MLP-expression in postnatal retinae. **A**: Quantitative real-time PCR of embryonic and postnatal rat retinae. Relative MLP expression is shown as fold change of the respective value at E18. MLP expression was increased at E20 compared to E18 and further increased significantly after birth. Highest MLP-expression was detected at P14 (a fold change of 6.6). MLP expression levels sharply decreased between P14 and P21 and reached a plateau afterwards. Comparison of relative expression: ***P≤0.05. **B**: Western Blot analysis of MLP protein in postnatal retinae (P0–P42). MLP levels increased slightly between P0 and P7 and peaked at P14. MLP levels were markedly decreased at P21 and were below detection in retinal lysates of P28 and older animals. MLP in heart muscle lysate served as positive control and β-actin as loading control.

Initial experiments revealed MLP expression in postnatal rat retinae. To assess the complete temporal pattern of MLP expression during retinal development, we examined embryonic, postnatal and adult rat retinae (E18-P42) using quantitative real-time PCR and western blot analysis. Real-time PCR revealed low expression levels of MLP in E18 and adult retinae (Fig. 1. A). Expression increased slightly in E20 retinae and was significantly elevated at P0 to P14, with peak expression at P14 (∼7 fold). Thereafter, MLP expression rapidly decreased, falling to levels comparable to embryonic stages. (Fig. 1 A).

To verify the mRNA expression data at the protein level, we also performed western blot analysis on retinal lysates from P0–P42 rats. Sparse MLP protein was detectable at P0 (Fig. 1. B), but MLP levels increased between P3 and P14. In accordance with the results of the real-time PCR analysis, MLP expression peaked at P14 and markedly decreased at P21. No MLP protein was detected in retinal lysates at P28, P35 or P42.

### MLP is Expressed in Cholinergic Amacrine Cells

**Figure 2:**
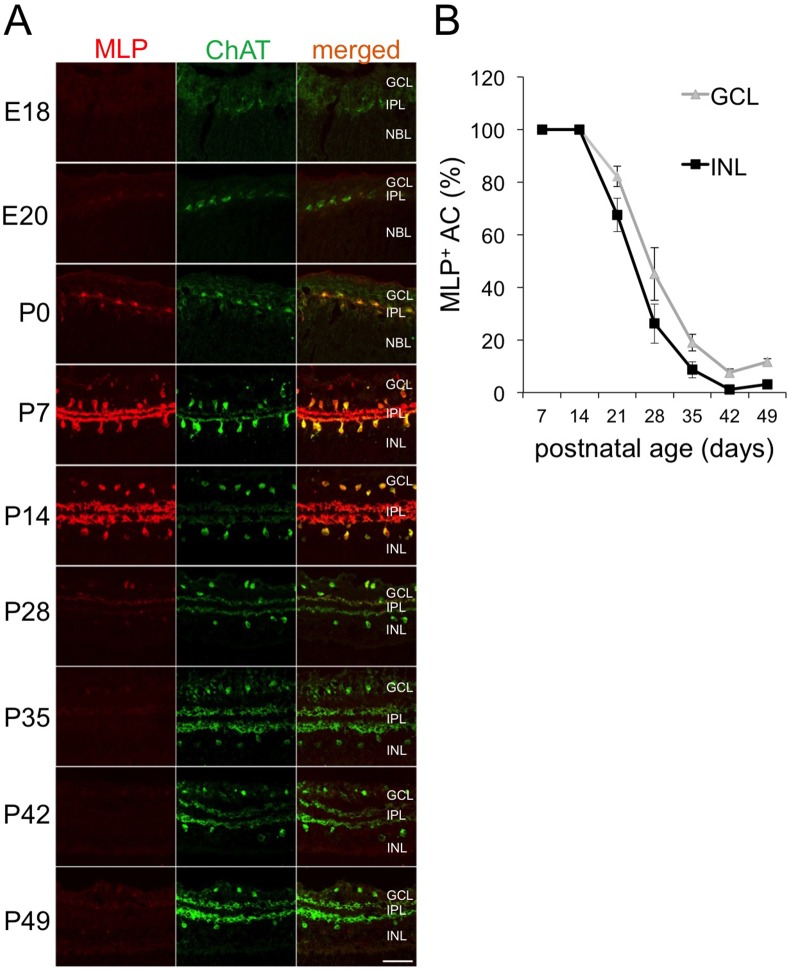
Expression of MLP in postnatal ChAT-positive AC. **A**: Confocal images were taken of E18, E20, P0, P7, P14, P28, P35, P42 and P49 retinae after co-staining with antibodies against ChAT and MLP. MLP expression becomes detectable at E20 in ChAT-positive AC located in the inner nuclear layer as well as ganglion cell layer. Levels reached a peak between P7 and P14. MLP expression dramatically decreases afterwards. GCL = ganglion cell layer, IPL = inner plexiform layer, INL = inner nuclear layer Scale bar: 50µm. **B**: Quantification of MLP-positive cholinergic AC in the inner nuclear layer (INL) and ganglion cell layer (GCL, displaced) of retinae of 7–49 days old rats. All cholinergic AC were positive for MLP at P7 and P14. Numbers of MLP-positive AC continuously decreased between P21 and P42. This attenuation proceeded slightly faster in the INL than in the GCL.

**Figure 3:**
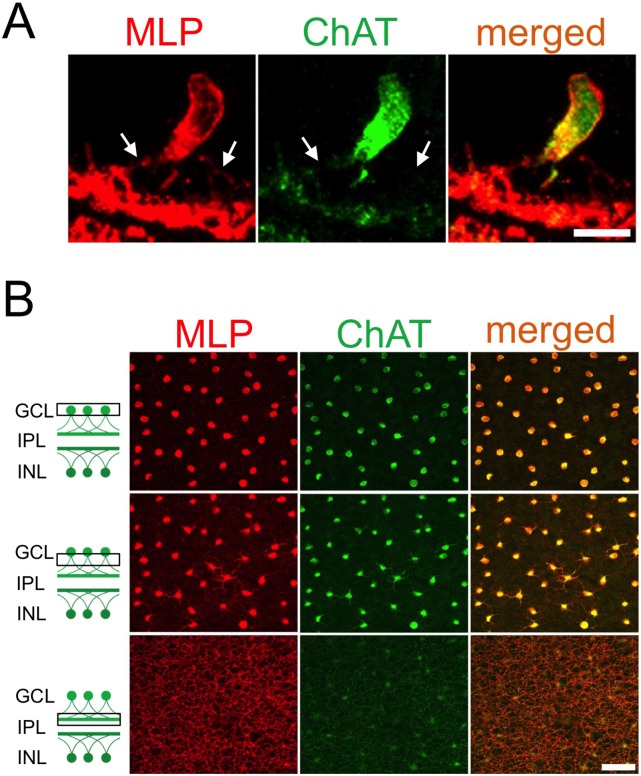
Subcellular localization of MLP in cholinergic AC. **A**: Confocal image of a MLP-positive cholinergic AC at P7, demonstrating MLP protein in the soma and the dendrites (arrows), but not in the nucleus. Scale bar: 10 µm. **B**: Confocal image of cholinergic AC in a retinal flatmount at P9, co-stained with antibodies against MLP and ChAT. The somas of all cholinergic amacrine cells as well as their proximal and distal dendrite segments are positive for MLP. Scale bar: 50 µm.

We next investigated the cellular localization of MLP in embryonic and postnatal retinae. Immunohistochemical staining of retinal cross-sections identified MLP-positive cells in the GCL and the INL of P14 retinae (Fig. 2 A). Co-immunostaining with an antibody against ChAT, a specific marker for cholinergic AC, revealed rigorous co-localization of ChAT and MLP staining, demonstrating that MLP expression is restricted to cholinergic AC only (Fig. 2 A, Fig. 3 B). MLP staining was detected on postnatal AC somas and dendrites whereas the nucleus remained spared (Fig. 3 A, Fig. 3 B). In addition, both sublaminae of the IPL containing dendrites of cholinergic AC from the GCL and the INL, respectively, were prominently immunoreactive for MLP.

We next analyzed the time course of MLP expression and quantified MLP-positive cholinergic AC in late embryonic and postnatal retinae. Slight MLP expression co-localizing with ChAT-positive cells was first detected at E20 (Fig. 2 A). The intensity of MLP immunoreactivity and number of MLP-positive cells substantially increased at P0 close to a still barely developed IPL. At P7 and P14, all cholinergic AC expressed MLP protein (Fig. 2 A, Fig. 2 B) and two subpopulations of MLP-positive cells located either in the GCL or the INL were clearly distinguishable. MLP staining markedly decreased in both subpopulations of cholinergic AC at P28 (Fig. 2A, Fig. 2 B). This reduction was slightly more pronounced in the INL compared to the GCL. On average, 45% of ChAT-immunoreactive cells of the GCL and 26% in the INL were still faintly MLP-positive at P28. Consistent with the Western-blot results, MLP remained weakly expressed in only very few cholinergic AC at P42 and P49 (Fig. 2A, Fig. 2 B).

### MLP is Expressed in Cultured Cholinergic AC

**Figure 4:**
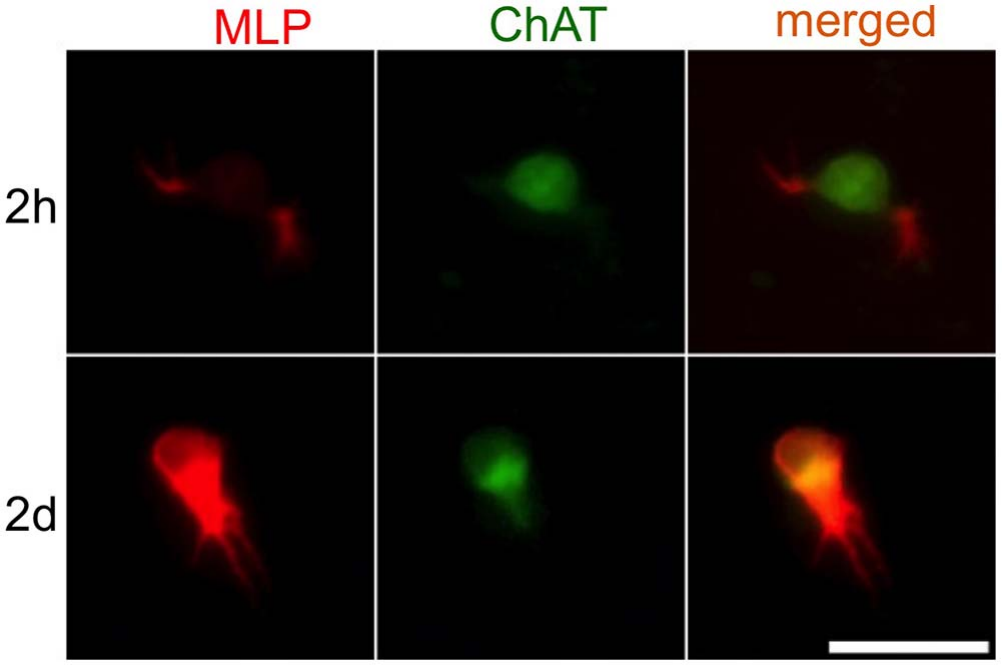
MLP-expression in cultured postnatal AC. Cultured cholinergic AC were obtained from the retina of a P1-rat. The cells were fixed and stained with antibodies against MLP and ChAT after 2 hours and 2 days in culture. After 2 hours, the cholinergic AC showed a week MLP expression in the soma. In sprouting dendrites, however, MLP-expression was more pronounced. After 2 days, the MLP levels dramatically increased in the soma as well as in the dendrites of the cholinergic AC. Scale bar: 25 µm.

We tested whether MLP-Expression can also be observed in cultured postnatal cholinergic AC. To this end cultures of cholinergic AC were prepared from retinae of rats at P1. MLP-expression was observed in small sprouting dendrites of cholinergic ACs after 2 hours in culture (Fig. 4). After 2 days MLP levels markedly increased in the somas and in the dendrites (Fig. 4). The dendrites, however, were not immunoreactive for ChAT. These data indicate that MLP is a suitable marker for outgrowing dendrites of cultured postnatal cholinergic AC.

## Discussion

MLP is a well-characterized protein, mainly expressed in heart tissue. It is involved in myogenesis and the organization of cytoskeletal structures in myocytes and has so far been regarded as muscle-specific [1], [4], [8]. The current study demonstrates to our knowledge for the first time that this protein is also markedly expressed in postnatal CNS tissue and therefore not restricted to muscle tissue. Western-blot analysis and quantitative PCR revealed a transient retinal MLP expression starting at E20, reaching a peak between P7 and P14 and absence in the adult retina. Immunohistochemical analysis confined MLP expression in cholinergic (ChAT-positive) AC. These neurons are one of at least 26 distinguishable AC subtypes with the ability to produce the neurotransmitter acetylcholine and are involved in the maturation of postnatal retina [19], [20] as well as motion sensation by the visual apparatus [27], [28].

In rodents, first AC are born at E8/E9, along with retinal ganglion cells (RGCs), horizontal cells and cone photoreceptors. AC genesis peaks at E16/E17, but proceeds at least until P5/P7 [29], [30]. Consistent with the data presented in the current study, cholinergic AC start to express choline acetyltransferase (ChAT) at around E18 and therefore almost simultaneously to the observed induction of retinal MLP-expression [15]. However, in contrast to the lifelong expression of ChAT, MLP is only expressed throughout the first 3 weeks after birth and absent in adult AC. During this postnatal time period AC form dendrites (E18), establish two well-separated cholinergic dendritic layers in the IPL (P3) [15] and make synapses on RGCs and bipolar cells (P3–P15) [31]. This process also timely overlaps with the emergence of spontaneous acetylcholine dependent waves of excitatory activity between P0 and P11 [32], which are important for the formation of synapses and the establishment of neural circuits between retinal neurons. The formation of specific synapses between cholinergic AC and direction-selective RGCs, known to be crucial for motion sensing, occurs in the second postnatal week and is therefore timely correlated with morphologic maturation of AC [22]. Maturation of cholinergic AC is eventually accomplished at postnatal day 15, the time of eye opening [15], [21] and of detected decrease of MLP expression, suggesting a potential functional involvement of this protein in this context. Future studies still have to investigate the potential role of MLP during AC development. To this end the morphology of AC or other retinal cells as well as the visual function of MLP deficient and wild-type mice could be compared with each other to identify potential differences.

In muscle tissue MLP is crucially involved in myogenesis and myocyte differentiation, requiring the localization of MLP in the nucleus, where it serves as transcriptional cofactor and modulates the expression of myocyte specific genes [8]. In addition, MLP is also located in the cytoplasm where it might promote the assembly of cytoskeletal proteins along actin-based filaments [4]. As MLP was only detected in the cytoplasm of AC it appears more likely that it is rather involved in the organization of cytoskeleton than regulation of gene expression in these neurons. In this context MLP may support the growth and stratification of dendrites or synapse formation. MLP has been shown to interact with a broad variety of proteins belonging to different functional classes in muscle tissue [33], [34]. In particular, interactions of MLP with α-actin [35], actin-binding proteins like cofilin 2 [2], spectrins (βI-spectrin) [5] or even metabolic enzymes like D-lactate dehydrogenase [36] were described. Therefore, the role of MLP in cholinergic AC might depend on the interaction with several partners. Further research is required to address these possibilities. Nevertheless, MLP expression can now be used as a novel marker for postnatal cholinergic AC.
