# Correction: Neuronal Expression of Muscle LIM Protein in Postnatal Retinae of Rodents

**DOI:** 10.1371/journal.pone.0109009

**Published:** 2014-09-30

**Authors:** The PLOS ONE Staff

There is an error in the legend for Figure 1, “Transient MLP-expression in postnatal retinae.” The complete, correct Figure 1 legend is:

**Figure 1:**
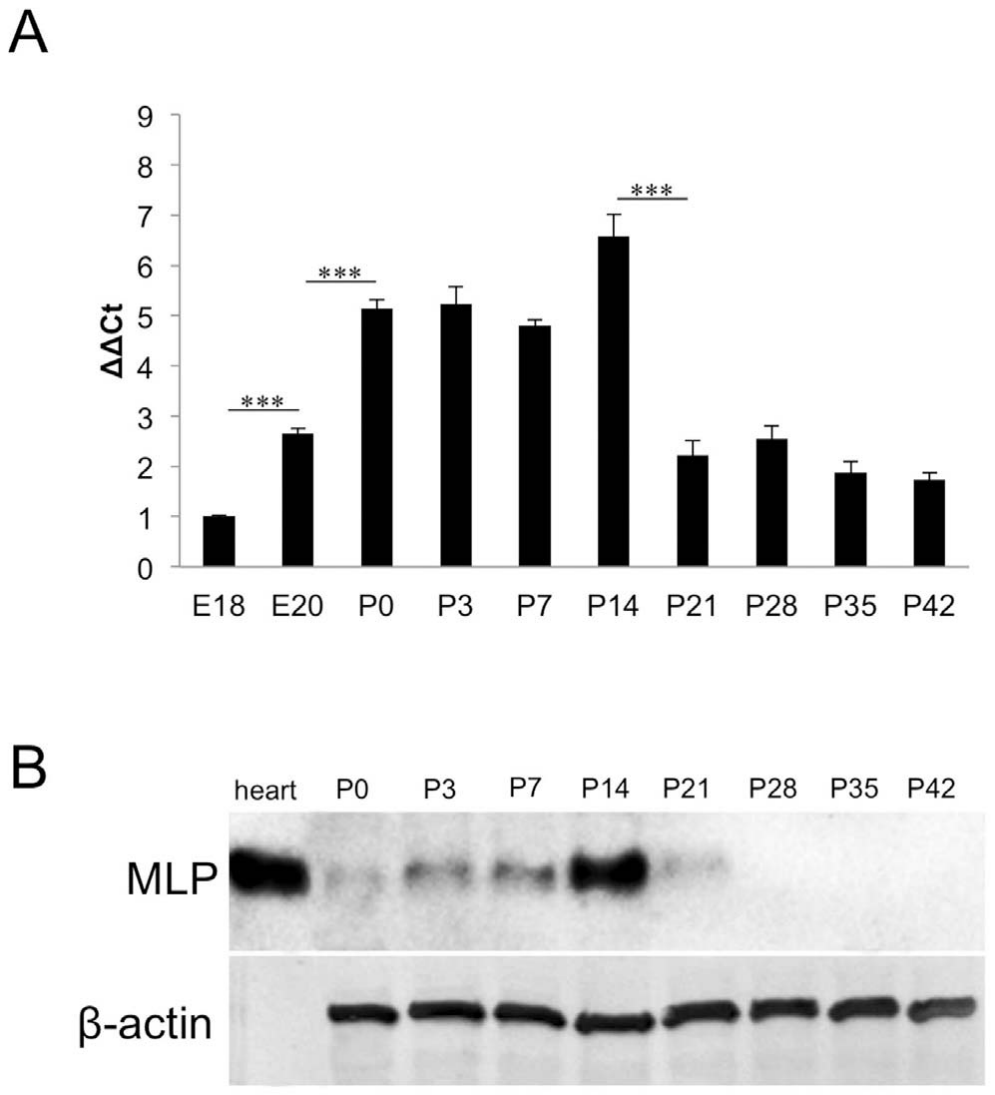
Transient MLP-expression in postnatal retinae. **A**: Quantitative real-time PCR of embryonic and postnatal rat retinae. Relative MLP expression is shown as fold change of the respective value at E18. MLP expression was increased at E20 compared to E18 and further increased significantly after birth. Highest MLP-expression was detected at P14 (a fold change of 6.6). MLP expression levels sharply decreased between P14 and P21 and reached a plateau afterwards. Comparison of relative expression: ***P≤0.001. **B**: Western Blot analysis of MLP protein in postnatal retinae (P0–P42). MLP levels increased slightly between P0 and P7 and peaked at P14. MLP levels were markedly decreased at P21 and were below detection in retinal lysates of P28 and older animals. MLP in heart muscle lysate served as positive control and β-actin as loading control.
